# Increased fMRI Sensitivity at Equal Data Burden Using Averaged Shifted Echo Acquisition

**DOI:** 10.3389/fnins.2016.00544

**Published:** 2016-11-23

**Authors:** Suzanne T. Witt, Marcel Warntjes, Maria Engström

**Affiliations:** ^1^Center for Medical Image Science and Visualization, Linköping UniversityLinköping, Sweden; ^2^Division of Cardiovascular Medicine, Department of Medical and Health Sciences, Linköping UniversityLinköping, Sweden; ^3^Department of Medical and Health Sciences, Linköping UniversityLinköping, Sweden

**Keywords:** fMRI, shifted echo, sampling rate, fast imaging, BOLD, EPI, temporal averaging, data burden

## Abstract

There is growing evidence as to the benefits of collecting BOLD fMRI data with increased sampling rates. However, many of the newly developed acquisition techniques developed to collect BOLD data with ultra-short TRs require hardware, software, and non-standard analytic pipelines that may not be accessible to all researchers. We propose to incorporate the method of shifted echo into a standard multi-slice, gradient echo EPI sequence to achieve a higher sampling rate with a TR of <1 s with acceptable spatial resolution. We further propose to incorporate temporal averaging of consecutively acquired EPI volumes to both ameliorate the reduced temporal signal-to-noise inherent in ultra-fast EPI sequences and reduce the data burden. BOLD data were collected from 11 healthy subjects performing a simple, event-related visual-motor task with four different EPI sequences: (1) reference EPI sequence with TR = 1440 ms, (2) shifted echo EPI sequence with TR = 700 ms, (3) shifted echo EPI sequence with every two consecutively acquired EPI volumes averaged and effective TR = 1400 ms, and (4) shifted echo EPI sequence with every four consecutively acquired EPI volumes averaged and effective TR = 2800 ms. Both the temporally averaged sequences exhibited increased temporal signal-to-noise over the shifted echo EPI sequence. The shifted echo sequence with every two EPI volumes averaged also had significantly increased BOLD signal change compared with the other three sequences, while the shifted echo sequence with every four EPI volumes averaged had significantly decreased BOLD signal change compared with the other three sequences. The results indicated that incorporating the method of shifted echo into a standard multi-slice EPI sequence is a viable method for achieving increased sampling rate for collecting event-related BOLD data. Further, consecutively averaging every two consecutively acquired EPI volumes significantly increased the measured BOLD signal change and the subsequently calculated activation map statistics.

## Introduction

It has long been known that increasing the acquisition rate of blood oxygen level dependent (BOLD) data results in more accurate measurements of the hemodynamic response function (HRF). Not only does theory demonstrate that MR signal is inversely proportional to the sampling rate, increased temporal sampling of the BOLD response, in particular, has been shown to result in measured signals that more closely match the predicted signal (Dilharreguy et al., 2003). Studies using both real and simulated fMRI data have also shown that the accuracy with which the peak of the HRF can be determined increases with decreased repetition time (TR; Miezin et al., 2000; MacCotta et al., 2001; Dilharreguy et al., 2003). Furthermore, if the width of the BOLD peak is narrower than the experimental TR, a loss of signal is inevitable. There has been a recent growth in the development of acquisition techniques aimed at increasing the temporal sampling of BOLD fMRI data including echo-volumar imaging (EVI; van der Zwaag et al., 2006; Rabrait et al., 2008; Witzel et al., 2008), inverse imaging (InI; Lin et al., 2006, 2010, 2012a,b), and multiplexed EPI (Feinberg et al., 2010). Beyond simply improving the accuracy with which the BOLD response can be measured, a number of additional benefits of increasing the temporal sampling of BOLD data have been highlighted, including reduced sensitivity to intra-scan head movement and better resolution of physiologic signal fluctuations (Posse et al., 2012; Smith et al., 2013). While there are a number of factors confounding the determination of an optimal TR with which to collect BOLD data including intra-subject variability, regional variability within the brain, experimental timings, and acquisition parameters, the results from these initial ultra-short TR fMRI studies all point toward the considerable benefits of collecting fMRI data with sampling rates of <1 s per volume (Feinberg et al., 2010; Posse et al., 2012; Lin et al., 2012a,b; Feinberg and Setsompop, 2013; Smith et al., 2013; Bhavsar et al., 2014; Chen et al., 2015; Todd et al., 2016).

A higher sampling rate is associated with an increased data burden, and there can be instances where prioritizing data set size and relative speed of offline analyses is preferred, particularly for task-based fMRI. Two recent studies investigating the suitability of EVI and InI imaging for fMRI applied moving average filters in an effort to further reduce the effects of physiologic noise and noted that this temporal smoothing resulted in a significant increase in the mean and maxima of the calculated activation map statistics (Lin et al., 2012a; Posse et al., 2012). These results would suggest that there might be some benefit in acquiring task-based fMRI data at a shorter TR and then employing some sort of temporal averaging scheme to achieve a smaller data set with an effectively slower TR than simply to opt to collect data at a slower TR from the outset. At the very least, the averaging of consecutively acquired volumes should ameliorate the reduced temporal signal-to-noise typically observed for ultra-short TR echo planar imaging (EPI) sequences (Feinberg et al., 2010; Posse et al., 2012; Feinberg and Setsompop, 2013; Smith et al., 2013).

**Figure 1:**
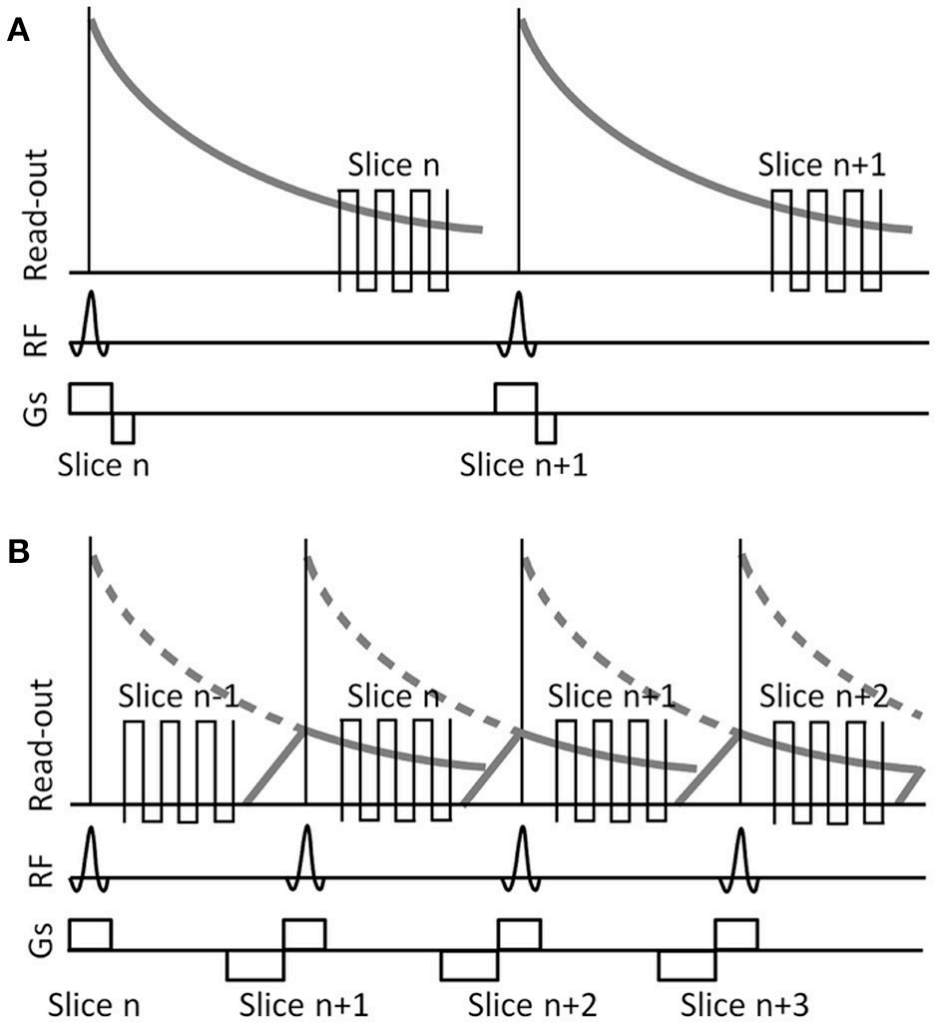
**Schematic illustrating slice excitation and read-out for a typical fMRI EPI sequence (A)** and a sequence incorporating a shifted-echo **(B)**. RF, radio frequency. Gs, slice selection gradient. In the typical fMRI EPI sequence data are acquired at a long TE, forcing a long repetition time TR between consecutive RF pulses. In the shifted-echo fMRI EPI sequence, the delay time between the end of the RF excitation and the start of the read-out is used to acquire another slice, effectively reducing the TR to half the time, while maintaining the same long TE for each slice. Gradient spoiling and rephrasing is used to control the signal strength in each slice.

Many of these new techniques developed to acquire EPI data with a significantly increased sampling rate require hardware, software, and non-standard data processing pipelines that may not be available to all researchers. One potential and readily available method to collect BOLD-EPI data at a TR of <1 s with an acceptable spatial resolution would be to incorporate the method of shifted echo into a standard multi-slice, gradient echo EPI sequence. The concept of shifted echo is schematically depicted in Figure 1. The transverse magnetization after a slice-selective RF pulse is purposely dephased to suppress signal from that slice during the time between the RF pulse and the typical echo time for fMRI experiments at TE = 40 ms. This allows measuring another slice during that time. The magnetization can be recalled by adding proper gradients prior to each RF pulse, effectively producing an echo time that is shifted by 1 TR (Moonen et al., 1992; Chung and Duerk, 1999). Pulse sequences incorporating a shifted echo typically store the excited magnetization along the transverse axis until spoiler gradients are used to recall it at a later TR interval. The use of shifted echo sequences for the acquisition of BOLD data is not new (Liu et al., 1993a,b; Duyn et al., 1994; Moonen et al., 1994; Chung and Duerk, 1999; Gibson et al., 2006; Chang et al., 2013; Ehses et al., 2015), however, these previous studies have, for example, achieved increased sampling rates at the expense of decreased spatial resolution and/or slice coverage (Gibson et al., 2006), prioritized spatial resolution at the expense of temporal resolution (Ehses et al., 2015), or used a non-standard multi-slice EPI sequence (Liu et al., 1993a; Duyn et al., 1994; Chang et al., 2013; Ehses et al., 2015).

The goal of any task-based fMRI experiment is ultimately to maximize the signal difference between task-related neural activity and some predefined baseline. As summarized above, both early and recent work into acquisition techniques for BOLD data suggest that the best method for maximizing the sensitivity to the relatively small signal change of the BOLD response is to significantly increase the rate at which the BOLD response is sampled. We propose to use a multi-slice gradient echo EPI sequence with shifted echo and sub-one second TR combined with temporal averaging of consecutively acquired volumes to produce EPI data with increased BOLD signal with a reduced data set size.

## Methods

### Reference EPI sequence

A standard single-shot gradient-echo EPI sequence that covered the whole brain with a TR of 1440 ms (referred to as REF in figures and tables) was used as a reference to which the results from the short TR shifted echo sequence and subsequent spatial averaged sequences could be compared. The reference sequence parameters were as follows: TR/TE = 1440/37 ms; flip angle = 72°; FOV = 230 × 230 mm^2^; voxel = 3.59 × 3.59 mm^2^; slice thickness (gap) = 4 (0.5) mm; matrix = 64; EPI factor [# k-space lines] = 31; slices = 28; SENSE factor 2. The EPI readout duration was 20.5 ms, resulting in a water-fat shift of 8.9 pixels (bandwidth = 48.8 Hz/pixel). The reference sequence did not include lipid suppression, which may have resulted in small chemical shift artifacts. However, since the lipid signal should have been relatively stable, or at least not changed in synchrony with the experimental task timings, there should have been little to no effect on the overall results.

### Shifted echo EPI sequence

A shifted echo was included in the reference EPI sequence described above effectively covering the whole brain with a TR of 700 ms (referred to as SHORT in figures and tables). The parameters for the shifted echo EPI sequence were identical to those of the reference scan apart from the flip angle [flip angle = 56°; FOV = 230 × 230 mm^2^; voxel = 3.59 × 3.59 mm^2^; slice thickness (gap) = 4 (0.5) mm; matrix = 64; EPI factor = 31; slices = 28; bandwidth = 48.8 Hz/pixel; SENSE factor = 2], with the addition of a shifted echo: TR/TE = 25/37 ms. To examine the effects of temporal averaging of this sequence to produce effective TRs more in line with sequences currently used in typical task-based fMRI studies, two variants of this shifted-echo sequence were created using the built in spatial averaging option on the scanner. For the first variant, the scanner was set to average every two consecutively acquired volumes to produce an EPI sequence with an effective TR of 1400 ms (hereafter referred to as AVG2 in text, figures, and tables). For the second variant, the scanner was set to average every four consecutively acquired volumes to produce an EPI sequence with an effective TR of 2800 ms (hereafter referred to as AVG4 in text, figures, and tables).

### Experimental design and data acquisition

Eleven right-handed participants were recruited through word-of-mouth. All participants reported to be in good general physical and mental health. Written informed consent was obtained according to the Declaration of Helsinki, and approval for the study was granted by the Regional Ethical Review Board in Linköping, Sweden (Dnr. 2015/198-31).

Participants completed four parallel runs of a simple, sparse event-related visual-motor task. During the task, participants were shown a series of shapes (circles, squares, and triangles) and instructed to press a button with their right index finger when shown a square, press a button with their right middle finger when shown a triangle, and to not respond at all when shown a circle. The task was designed to be a slow Go/No-go type response inhibition task that would be simple enough for participants to perform accurately with minimal practice but challenging enough to maintain the participants' focus for the duration of the task. The design of the task also ensured that activation would be observed throughout the brain. A short practice run was completed prior to scanning to ensure that all participants understood the task instructions. Each shape was presented for 500 ms six times in pseudo-random order during each run of the task, where the inter-stimulus interval varied between 19 and 22 s and total task length was ~5 min 20 s. The pairing of each task run with each EPI sequence and the order in which the EPI sequences were acquired were randomized across subjects. The task was presented using Superlab 5 (Cedrus Inc., San Pedro, CA) via MRI compatible goggles (Resonance Technology Inc., Northridge, CA). Participant responses were collected using a Lumina fiber optic response pad (Cedrus Inc., San Pedro, CA). All scans were acquired using a 32 channel coil on a Philips Ingenia 3T scanner (Philips Healthcare, Best, The Netherlands) located at the Center for Medical Image Science and Visualization, Linköping University Hospital, Linköping, Sweden.

### FMRI data analysis

**Figure 2:**
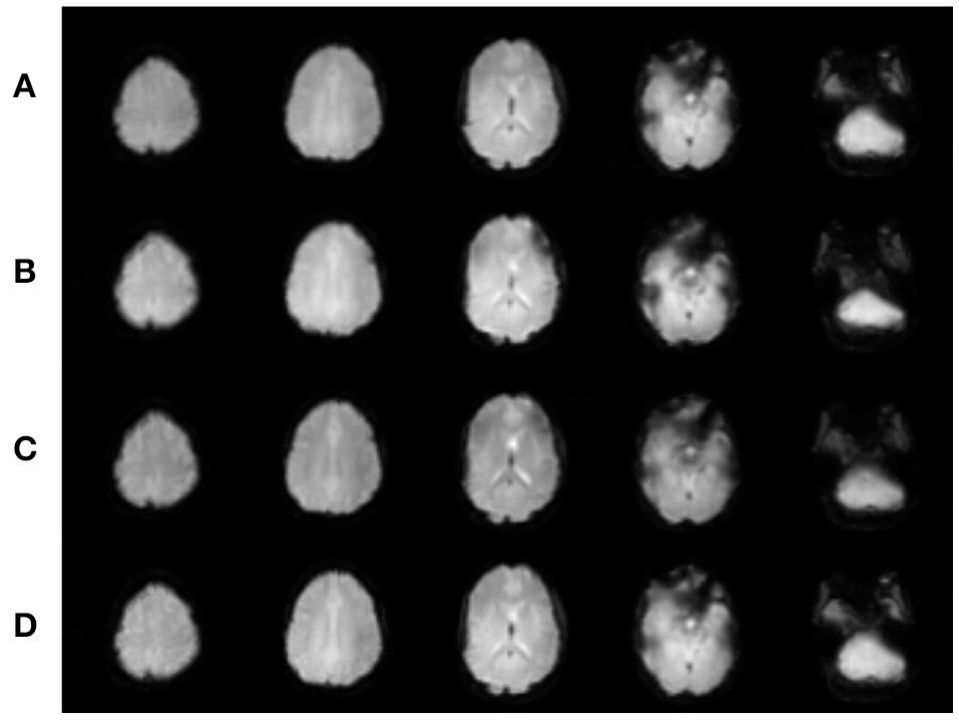
**Realigned images for each EPI sequence for one participant. (A)** SHORT, TR = 700 ms; **(B)** AVG2, TR_effective_ = 1400 ms; **(C)** AVG4, TR_effective_ = 2800 ms; **(D)** REF, TR = 1440 ms. Slices were created using Mango (http://ric.uthscsa.edu/mango/; Jack L. Lancaster and Michael J. Martinez).

The data were preprocessed and analyzed using SPM12 (The Wellcome Trust Centre for Neuroimaging, University College London, London, UK). All participants' images were separately realigned, where the resulting translation and rotation parameters were individually examined to ensure that no participant had either significant head motion exceeding the dimensions of one voxel in any direction or had head motion that was significantly correlated with experimental timings. No data were excluded for either reason. Each participant's realigned EPI images were then coregistered with his/her own T1 anatomical scan. Spatial normalization into Montreal Neurologic Institute (MNI) space was initially performed on each participant's T1 anatomical scan, and these spatial normalization parameters were then applied to each respective functional data set. The spatially normalized images were smoothed with an 8 mm FWHM Gaussian kernel. Selected slices of the realigned EPI images for one participant for each of the four EPI sequences are shown in Figure 2.

**Figure 3:**
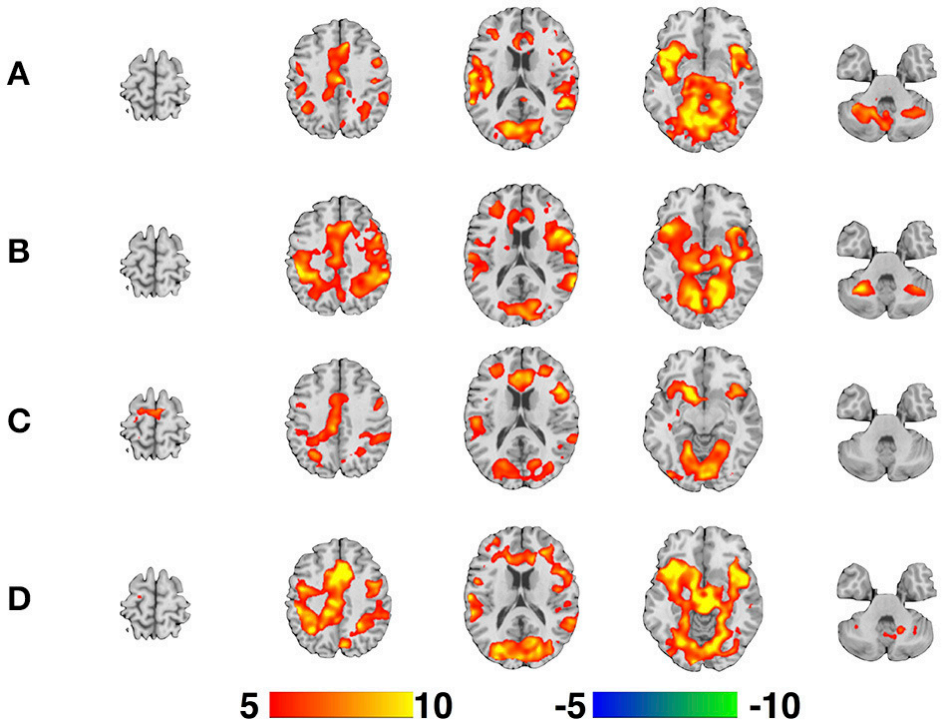
**One-sample *t*-test maps for each of the four EPI sequences. (A)** SHORT, TR = 700 ms; **(B)** AVG2, TR_effective_ = 1400 ms; **(C)** AVG4, TR_effective_ = 2800 ms; **(D)** REF, TR = 1440 ms. Color bar is given in terms of *T*-statistic, and maps are thresholded at *p* < 0.05, Family-Wise Error corrected. Slices were created using Mango (http://ric.uthscsa.edu/mango/; Jack L. Lancaster and Michael J. Martinez).

The regressors for each of the four parallel task runs were derived by extracting the onset timings for the visual presentation of the circle trials and the motor responses of the square and triangle trials and modeled using a synthetic HRF. The functional imaging data for each participant for each EPI sequence were modeled individually and included a single regressor that included all trials. The six motion-correction parameter estimates (x, y, and z displacements and pitch, roll, and yaw rotations) were included as covariates of no interest to statistically control for signal change related to head motion. A high-pass filter (cut-off period = 128 s) was incorporated into the model to remove low-frequency signals. A contrast image corresponding to the main effects of task performance was created and represented brain activity relative to an “implicit baseline” of unmodeled variance. Group level activation maps for each EPI sequence were calculated as a one-sample *t*-test across all 11 subjects (Figure 3).

### Temporal signal-to-noise calculation

To calculate the temporal signal-to-noise (tSNR), the realigned data were temporally smoothed voxel-wise with a Gaussian curve with a width of 10 TRs. This temporally smoothed data were then subtracted from the original realigned data to remove all long-term fluctuations in the data without any assumptions on specific frequencies. The data resulting from this subtraction contains only the high-frequency noise. The temporal standard deviation of this high-frequency noise data was calculated, and the whole-brain average was used as our metric for tSNR.

### Peri-stimulus timing histogram curve calculation

To examine the effects that both the inclusion of a shifted-echo and the spatial averaging of consecutively acquired EPI volumes may have had on the shape of the measured BOLD response, peri-stimulus timing histogram (PSTH) curves were created using MarsBar (release 0.44; Brett et al., 2002). The PSTH were converted to percent signal change by MarsBar using the same general procedure described below in Section BOLD Percent Signal Change Calculation, such that the resulting curves could be displayed in terms of units of percent signal change of the de-scaled beta weights. The PSTH curves were extracted for 20 s windows for each participant from the BOLD signal averaged across 8 mm spherical ROIs positioned at the peak stereotactic coordinates for four task-related regions-of-interest: primary visual cortex (V1: x = −2; y = −70; z = −4), left primary motor cortex (LM1: x = −40; y = −32; z = 52), supplementary motor area (SMA: x = −2; y = 2; z = 56), and the dorsal anterior cingulate cortex (ACC: x = −2; y = 26; z = 24). The PSTH curves represent the HRF collapsed across all task trials and averaged across all 11 participants. To further examine the variability in regional BOLD sensitivity with respect to the four sequences, the average percent signal change was extracted for each of the four 8 mm spherical ROIs described above.

### BOLD percent signal change calculation

The general procedure laid out by Luo and Nichols (2003) was used to calculate the percent signal change of the beta weight values determined during the general linear model (GLM) analysis such that they could be compared across the different EPI sequences. This procedure assumes the SPM approach to specifying the GLM and includes three scaling factors: (1) the peak value in the design matrix, (2) the normalization by a baseline value, and (3) the sum of the positive terms in the contrast vector. The third scaling factor is only applicable for studies using a subtraction paradigm and was not used in the present study. The percent signal change values for each EPI sequence were then calculated by multiplying the beta weight image for the main effects of task performance by the quotient of dividing the peak value in the design matrix by the whole brain average beta weight of the constant term in the design matrix. The percent signal change calculations were performed using the ImCalc tool in SPM12.

### Statistical analyses

Repeated measures ANOVAs were performed for the signal change values calculated for each EPI sequence. Initially, the ANOVA for signal change was performed on the average value extracted for each subject from an ROI mask comprised of task-related ROIs typically reported to be activated during response inhibition tasks (Buchsbaum et al., 2005; Simmonds et al., 2008; Criaud and Boulinguez, 2013) and selected from the FSL Harvard-Oxford Atlas (Frazier et al., 2005; Desikan et al., 2006; Makris et al., 2006; Goldstein et al., 2007). This ROI mask included ACC, right posterior supramarginal gyrus, left post-central gyrus, bilateral frontal operculum, bilateral thalamus, bilateral intracalcarine cortex, and bilateral SMA. Secondly, to determine whether any perceived benefits of either using an ultra-short TR or temporal averaging were confined solely to task-related regions or were generally seen across the whole brain, the repeated-measures analysis for signal change was performed on the average value extracted for each subject from the entire FSL Harvard-Oxford Atlas. Given the relatively small number of participants, omnibus significance was determined at *p* < 0.5. *Post-hoc* results were determined to be significant at *p* < 0.05, Bonferroni corrected for comparing across the four EPI sequences. All statistical analyses were performed using SPSS v22 (IBM Corporation, Armonk, NY).

### Supplemental analyses

Since most fMRI studies typically report results in terms of Student's *t*-statistics, the statistical analyses performed on the BOLD signal change values were repeated for the Student's *t*-statistics estimated during the GLM analysis for both the ROI mask and the full FSL Harvard-Oxford atlas. As these analyses were supplemental, *post-hoc* results were determined to be significant at *p* < 0.1, uncorrected.

## Results

### Temporal signal-to-noise ratio

**Figure 4:**
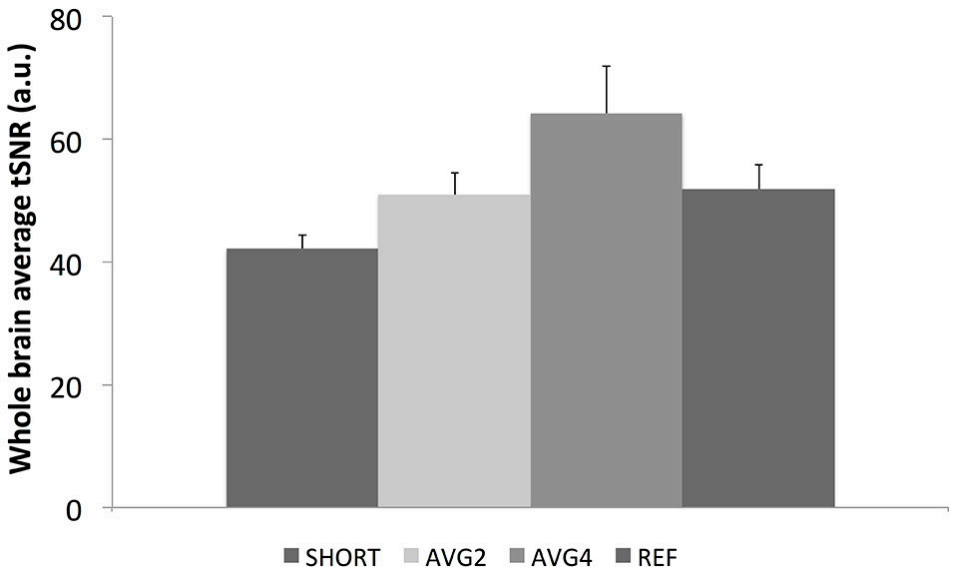
**Whole-brain average tSNR values for each EPI sequence**. SHORT, TR = 700 ms; AVG2, TR_effective_ = 1400 ms; AVG4, TR_effective_ = 2800 ms; REF, TR = 1440 ms.

The whole-brain average tSNR results for each EPI sequence averaged across all 11 participants are shown in Figure 4. The SHORT sequence had the lowest tSNR. The AVG2 and the reference sequences had approximately the same tSNR, while the AVG4 sequence exhibited the largest tSNR.

### Peri-stimulus timing histogram curves

**Figure 5:**
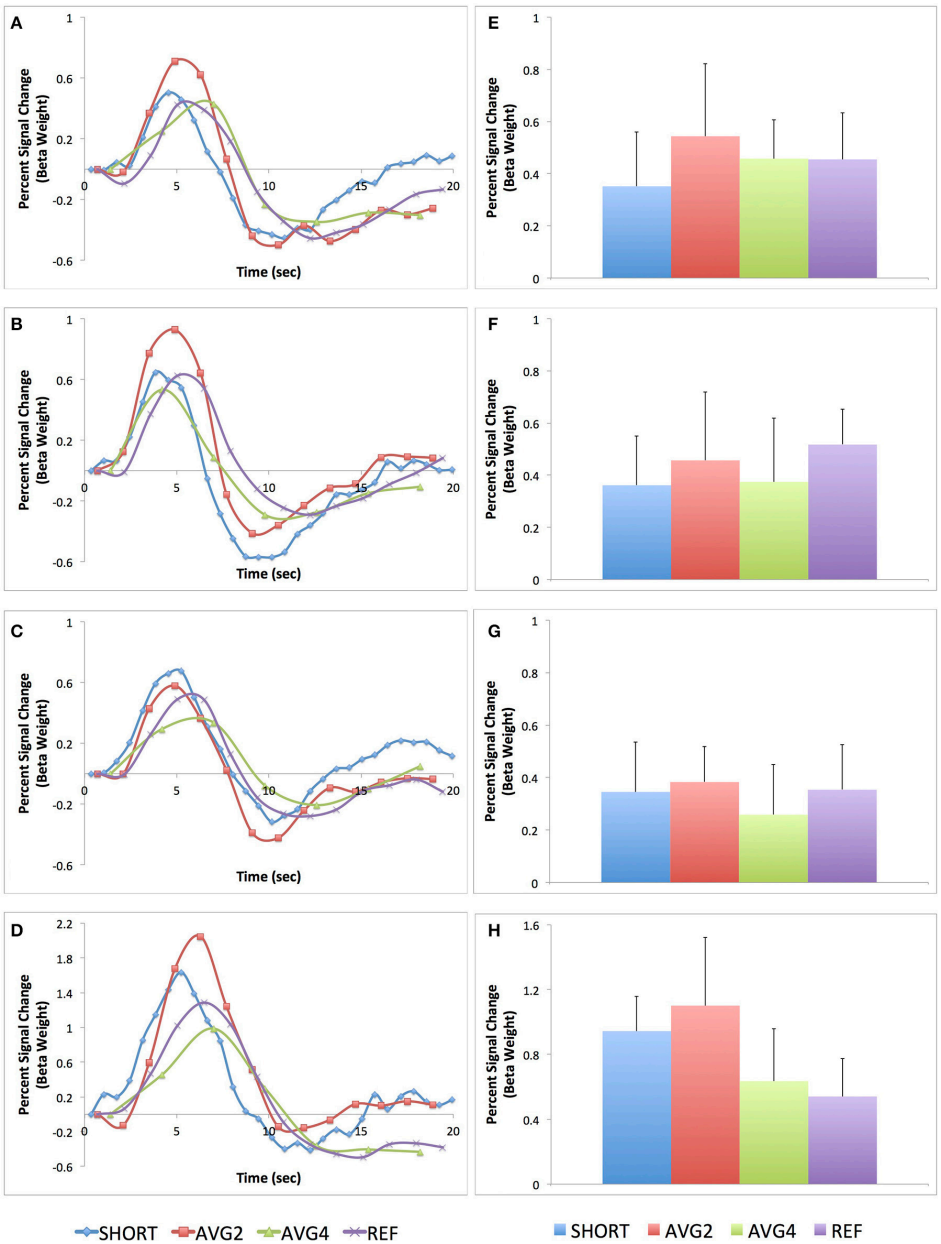
**Peri-stimulus histogram timing curves and average percent signal change for each of the four EPI sequences for four task-related regions-of-interest. (A)** PSTH curve for anterior cingulate cortex; **(B)** PSTH curve for supplementary motor area; **(C)** PSTH curve for left primary motor cortex; **(D)** PSTH curve for medial primary visual cortex; **(E)** Average percent signal change for anterior cingulate cortex; **(F)** Average percent signal change for supplementary motor area; **(G)** Average percent signal change for left primary motor cortex; **(H)** Average percent signal change for medial primary visual cortex. Both the curves and average percent signal change values are plotted in terms of percent signal change of the beta weight. Data from the SHORT sequence (TR = 700 ms) are plotted in blue, AVG2 sequence (TR_effective_ = 1400 ms) in red, AVG4 sequence (TR_effective_ = 2800 ms) in green, and REF sequence (TR = 1400 ms) in purple.

The average peri-stimulus timing histogram curves across all subjects are shown in Figures 5A–D. The average percent signal change for these same four regions are shown in Figures 5E–H. Both the PSTH curves and the measures of average percent signal change indicate that there is some regional variability in terms of sampling rate and regional BOLD sensitivity. Generally, though, the AVG2 sequence appeared to yield the highest sensitivity across the most brain regions.

### Task relevant ROIs

#### BOLD signal change

**Figure 6:**
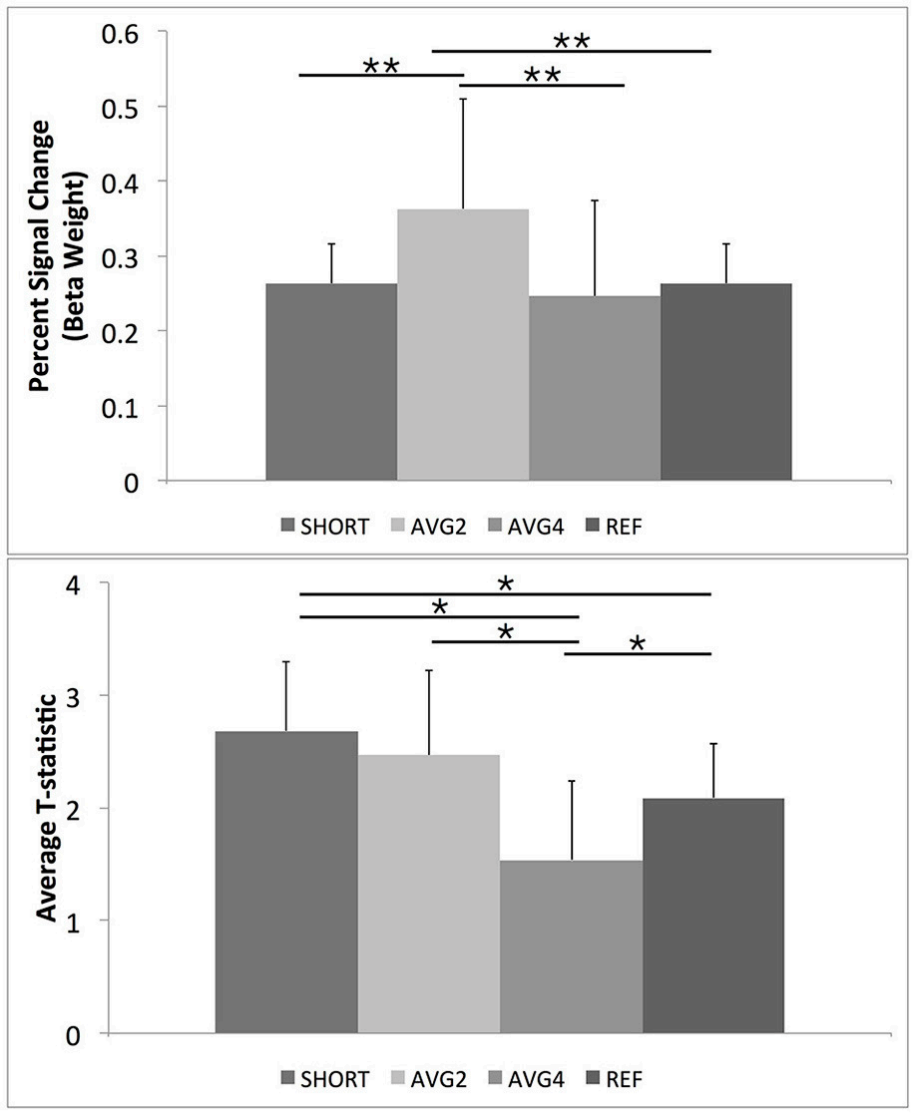
***Post-hoc* results for repeated measures ANOVAs for BOLD percent signal change and GLM-calculated *T*-statistics for the ROI mask containing only task-related regions extracted from the FSL Harvard-Oxford Atlas**. As described in the text, SHORT, TR = 700 ms; AVG2, TR_effective_ = 1440 ms; AVG4, TR_effective_ = 2800 ms; REF, TR = 1440 ms. Percent signal change values were calculated from the beta weights estimated during the GLM. ^**^Indicates a *post-hoc* difference at *p* < 0.05, Bonferroni corrected for comparing across the four different EPI sequences. ^*^Indicates a *post-hoc* difference at *p* < 0.05, uncorrected.

A trend-level omnibus repeated-measures effect was noted when comparing the calculated percent signal change across all sequences [*F*_(2, 9)_ = 3.41, *p* < 0.08]. Planned comparisons revealed that the AVG2 sequence had a significantly higher percent signal change than both the SHORT and AVG4 sequences. No other significant *post-hoc* comparisons were noted. A significant within-subjects effect was also noted [*F*_(3, 30)_ = 4.2, *p* < 0.03], indicating that this pattern of results was held across all subjects. Results are summarized in Table 1 and Figure 6.

**Table 1:** ***Post-hoc* results for the BOLD signal change and GLM-calculated *T*-statistics for the ROI mask containing only task-related regions**.

**Measure**	**SHORT**	**AVG2**	**AVG4**	**REF**	***Post-hoc***
Percent signal change (Beta Weight)	0.26 ± 0.05	0.36 ± 0.15	0.25 ± 0.12	0.26 ± 0.05	AVG2 > SHORT, AVG4, REF^**^
*T*-statistic	2.69 ± 0.61	2.47 ± 0.76	1.54 ± 0.67	2.09 ± 0.49	SHORT > AVG4^*^
					AVG2 > AVG4^*^
					REF > AVG4^*^

*As described in the text, SHORT, TR = 700 ms; AVG2, TR_effective_ = 1400 ms,; AVG4, TR_effective_ = 2800 ms; REF, TR = 1440 ms*.

** *Indicates a post-hoc difference at p < 0.05, Bonferroni corrected for comparing across the four different EPI sequences*.

* *Indicates a post-hoc difference at p < 0.05, uncorrected*.

#### Supplemental results on student's *t*-statistic

The supplemental repeated-measures ANOVA of *t*-statistic found significant omnibus effects [*F*_(3, 8)_ = 4.9, *p* < 0.03]. *Post-hoc* comparisons revealed that the AVG4 sequence was found to have a significantly lower average *t*-statistic than the remaining three sequences, while the AVG2 sequence was found to have a significantly higher *t*-statistic than the reference sequence. Again, a significant within-subjects effect was observed [*F*_(3, 30)_ = 7.5, *p* < 2.3 × 10^−3^], indicating that this general pattern of *post-hoc* results was preserved across all subjects. These supplemental results are summarized in Table 1, Figure 6.

### Whole brain

#### BOLD signal change

**Figure 7:**
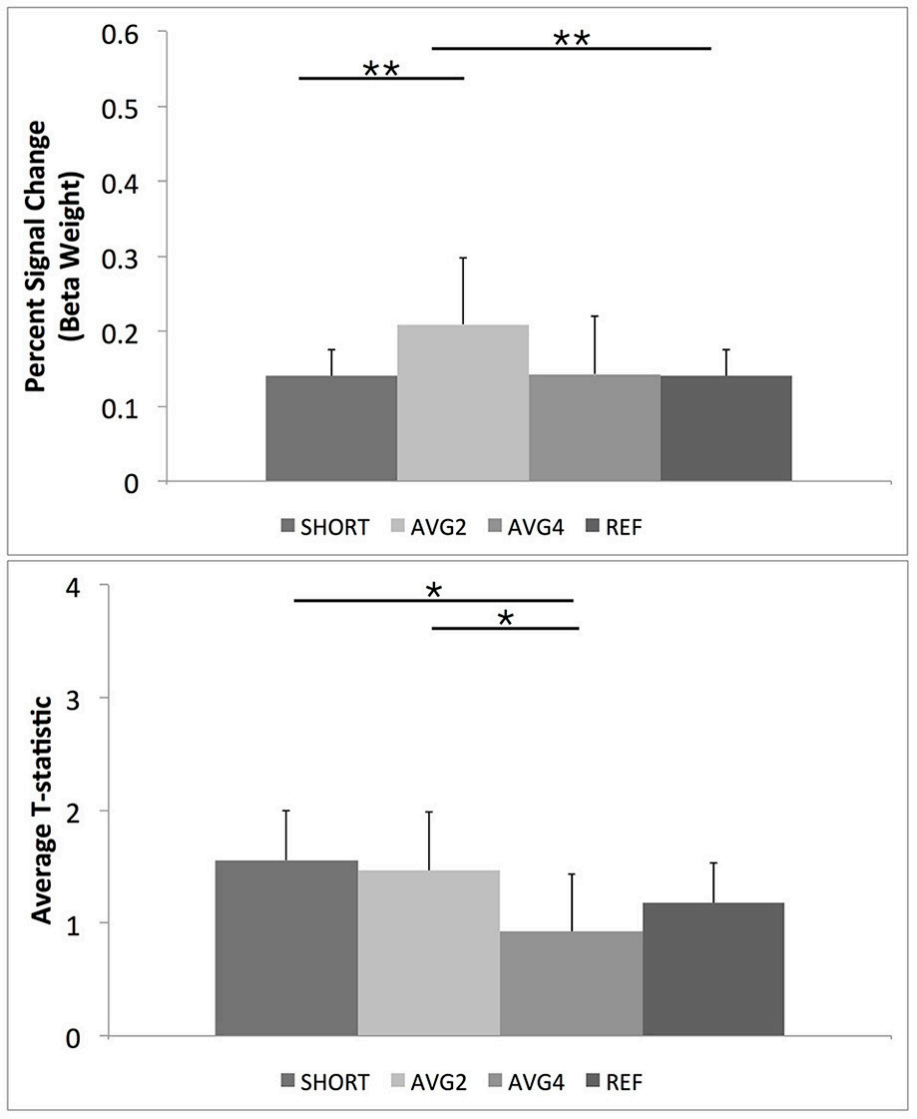
***Post-hoc* results for repeated measures ANOVAs for BOLD percent signal change and GLM-calculated *T*-statistics for the full FSL Harvard-Oxford brain atlas**. As described in the text, SHORT, TR = 700 ms; AVG2, TR_effective_ = 1440 ms; AVG4, TR_effective_ = 2800 ms; REF, TR = 1400 ms. Percent signal change values were calculated from the beta weights estimated during the GLM. ^**^Indicates a *post-hoc* difference at *p* < 0.05, Bonferroni corrected for comparing across the four different EPI sequences. ^*^Indicates a *post-hoc* difference at *p* < 0.05, uncorrected.

The repeated-measures results for comparing the percent signal change across the four sequences for the whole brain followed the same pattern as with the ROI mask results. Overall trend-level omnibus [*F*_(2, 9)_ = 3.6, *p* < 0.07] and significant within-subjects [*F*_(3, 30)_ = 3.4, *p* < 0.031] effects were noted. Also, planned comparisons showed that the AVG2 sequence had a significantly larger average signal change across the brain than both the shifted-echo and reference sequences. There were also statistic trends (*p* < 0.1) for the AVG2 sequence to have larger average percent signal change than the AVG4 sequence. Results summarized in Table 2, Figure 7.

**Table 2:** ***Post-hoc* results for repeated measures ANOVAs for BOLD signal change and GLM-calculated *T*-statistics for the full Harvard-Oxford brain atlas**.

**Measure**	**SHORT**	**AVG2**	**AVG4**	**REF**	***Post-hoc***
Percent signal change (Beta Weight)	0.14 ± 0.04	0.21 ± 0.09	0.143±0.07	0.14 ± 0.04	AVG2 > SHORT, REF^**^
*T*-statistic	1.56 ± 0.44	1.46 ± 0.52	0.93±0.48	1.18 ± 0.35	SHORT, AVG2 > AVG4^*^

*As described in the text, SHORT, TR = 700 ms; AVG2, TR_effective_ = 1400 ms; AVG4, TR_effective_ = 2800 ms; REF, TR = 1440 ms*.

** *Indicates a post-hoc difference at p < 0.05, Bonferroni corrected for comparing across the four different EPI sequences*.

* *Indicates a post-hoc difference at p < 0.05, uncorrected*.

#### Supplemental results on student's *t*-statistic

The supplemental repeated-measures ANOVA of the resulting t-statistic across the whole brain found a trend level omnibus effect [*F*_(3, 8)_ = 2.3, *p* < 0.1]. *Post-hoc* tests revealed that the AVG4 sequence had lower average *t*-statistic across the whole brain than both the AVG2 and reference TR sequences. There was also a trend for the AVG4 to have lower average *t*-statistic than the reference sequence (*p* < 0.1). Again, a significant within-subjects effect indicated that this pattern of *post-hoc* results was observable across all subjects [*F*_(3, 30)_ = 4.9, *p* < 0.02]. These supplemental results are summarized in Table 2, Figure 7.

## Discussion

We present data comparing three variations of an EPI sequence incorporating a shifted echo to achieve sub-one second TR with a standard multi-slice EPI sequence with a typical TR. All four EPI sequences had acceptable whole-brain average tSNR, were able to detect the BOLD signal, and produced acceptable group-level activation maps. As described more fully below, we specifically noted that the short TR sequence was preferable due to increased accuracy in measuring the hemodynamic response and increased reported *T*-statistics for the resulting task-based activation maps. We further noted that in instances where data burden may be an issue, averaging every two consecutively acquired EPI volumes resulted in increased tSNR and measured BOLD signal change without sacrificing the accuracy with which the HRF could be determined compared with the short TR sequence. Results from the reference TR and AVG4 sequences furthered confirmed that BOLD fMRI data should be acquired at a sampling rate of no more than 1 s per volume.

The peri-stimulus timing histogram curves indicated that the short TR sequence and the AVG2 sequence were able to produce estimates of the HRF with similar accuracy in terms of overall shape (e.g., pre-stimulus dip, peak, and post-stimulus return to baseline) as the reference sequence. For the four brain regions examined, both the short TR and AVG2 sequences did have higher peak values than the reference scan, though. Given the relatively short rise time, particularly for event-related experimental designs, the reference sequence appeared to sample too sparsely, and hence to a certain extent missed the peak of the BOLD response. Additionally, averaging across four consecutive volumes, as was performed for the AVG4 sequence, resulted in a flattening of BOLD response, most likely due to averaging the volume acquired during the peak of the BOLD response with those acquired during the rise and the return to baseline. As briefly described in the Results, we did observe some regional variation in the relationship between measured signal change and TR. The results, though, from both the PSTH curves and the averaged percent signal change from the same four spherical ROIs indicated that, in general, both the short TR and AVG2 sequences were consistently better able to detect to changes in the BOLD signal compared with either the AVG4 or reference sequences.

In line with the PSTH curve results, we noted that the AVG2 sequence gave significantly increased signal change compared with reference scan, suggesting that acquiring at a faster TR and averaging up to a slower TR yielded better differentiation of BOLD signal from the baseline noise than simply acquiring at the slower TR. There was also a statistical trend for AVG2 to yield higher percent signal change than the short TR scan, indicating that averaging of consecutively acquired EPI volumes resulted in improved fMRI signal compared with simply accelerating the acquisition speed. However, it was no longer apparent when comparing the average *T*-statistics for the AVG2 and the short TR sequences. As T-statistic is necessarily dependent on the sample size, having twice as many samples in the short TR data as with the AVG2 data resulted in nearly equivalent T-statistics despite the AVG2 scan having significantly higher measured percent signal change. This suggests that the previously published data that demonstrate that task-based fMRI studies would benefit from increased EPI acquisition speed is nominally correct (Feinberg and Setsompop, 2013; Bhavsar et al., 2014; Chen et al., 2015; Todd et al., 2016), but this benefit appears to be achieved solely due to the increased number of acquired volumes rather than any observed increase in BOLD signal afforded by the short TR EPI sequence. Our work shows that a higher sampling rate must not necessarily be associated with a larger data volume, rather, it suggests that the sampling rate should be as high as possible and may be used in conjunction with temporal averaging to achieve a reasonable TR to prevent an excessive data set size.

As stated above, the apparent advantages of averaging consecutively acquired EPI volumes diminished when increasing the number of averaged volumes from two to four. It is unclear whether this could be ameliorated by starting at an even lower TR, or if simply averaging across four (or more) consecutive scans effectively flattens out voxel-wise signal changes associated with the BOLD response, as indicated by the PSTH curves. This result was observed in spite of the AVG4 sequence exhibiting much higher whole-brain average tSNR than the other three sequences. Additionally, increasing the number of consecutively acquired volumes to be averaged necessarily reduces the number of samples by a commensurate amount, such that the increase in measured BOLD signal would need to be much larger than observed to counteract the loss in statistical power.

As with any MRI acquisition technique, there are several limitations to the above-proposed method. Unlike the newer multi-band methods, incorporation of a shifted echo into a standard multi-slice EPI sequence does not appear to simultaneously allow for both sub-one second TR and high spatial resolution (e.g., 1 mm isotropic). Additionally, the inclusion of averaging of consecutively acquired EPI volumes may not be ideal in subject populations where increased head movement might be a factor. Nor would the inclusion of averaging of consecutively acquired EPI volumes appear to be beneficial for resting-state studies, as effectively decreasing the sampling rate will decrease the ability to separate physiologic noise from the low frequency resting-state brain fluctuations. We also only tested this method using an event-related paradigm, so it remains to be demonstrated whether this technique would result in similar improvements in measured signal change for fMRI experiments employing block designs. Additionally, future studies may wish to examine whether further optimizing the shifted echo EPI sequence (e.g., including lipid suppression, prospective motion correction, etc.) and/or the use of different averaging schemes may improve upon the results presented here. These limitations aside, incorporating a shifted-echo into a standard multi-slice EPI sequence combined with averaging of every two consecutively acquired volumes would appear to offer a readily implemented method to acquire event-related task-based BOLD fMRI data with increased signal change in a more conventionally sized data set.

## Conclusions

We have confirmed that incorporating a shifted-echo into a standard multi-slice gradient-echo EPI sequence presents a feasible method for collecting event-related BOLD weighted fMRI data with sub-one second TR and an acceptable spatial resolution. Additionally, we further demonstrated that averaging consecutively acquired EPI volumes results in a significant increase in measured event-related BOLD signal compared with that measured using either an EPI sequence with short TR or an EPI sequence with a longer TR, maintaining a manageable data set size.

## Author contributions

SW designed the study, collected, and analyzed all data, and wrote the manuscript. MW designed the study, implemented the shifted-echo EPI sequence, assisted with data analysis, and participated in the preparation of the manuscript. ME assisted with study design and participated in the preparation of the manuscript.

### Conflict of interest statement

The authors declare that the research was conducted in the absence of any commercial or financial relationships that could be construed as a potential conflict of interest.
